# Accuracy and reproducibility of shear wave elastography according to the size and elasticity of lesions: A phantom study

**DOI:** 10.1097/MD.0000000000031095

**Published:** 2022-10-14

**Authors:** Harim Kim, Haejung Kim, Boo-Kyung Han, Ji Soo Choi, Eun Sook Ko, Eun Young Ko

**Affiliations:** a Department of Radiology and Center for Imaging Science, Samsung Medical Center, Sungkyunkwan University School of Medicine, Gangnam-gu, Seoul, South Korea.

**Keywords:** accuracy, breast elastography, reproducibility, shear wave elastography, ultrasonography

## Abstract

While the extrinsic factors affecting reproducibility of shear wave elastography (SWE) have been well documented, there are few resources assessing intrinsic characteristics of the lesion affecting the reproducibility and accuracy of SWE. In this regard, this study aimed to evaluate the accuracy of measured elasticity and the reproducibility of SWE according to the lesion size and stiffness. Two breast radiologists examined 20 targets of 4 different levels of stiffness and 5 different sizes (2.5, 4, 7, 11, and 18 mm) in a customized elasticity phantom. The B-mode image, color elastography image, and kPa measurement were obtained twice by each examiner with a 1-week interval. Inter- and intra-observer reproducibility and the accuracy of measured kPa were analyzed using intraclass correlation coefficient (ICC) and Bland-Altman analysis. Subgroup analysis was run to evaluate the effect of lesion size and stiffness on the reproducibility and accuracy of measured kPa. Inter- and intraobserver reproducibility for measuring kPa showed excellent agreement (ICC: 0.9742 and 0.9582; ICC: 0.9932 and 0.9294). The size and stiffness of the targets did not affect reproducibility. The overall accuracy of measured kPa was very high (ICC: 0.8049). In the subgroup analysis, targets that were ≤4 mm in size showed lower accuracy (ICC: 0.542), whereas targets that were 7 and 11 mm in size showed higher accuracy (ICC: 0.9832 and 0.9656, respectively). SWE shows excellent reproducibility regardless of lesion size or stiffness in phantom targets. The accuracy of measured kPa is high in lesions that are 7 and 11 mm in size but is low in lesions that are ≤4 mm in size.

## 1. Introduction

In contrast to conventional B-mode ultrasonography (US) imaging, elastography offers additional diagnostic information about intrinsic tissue properties. For this reason, elastography is used for various purposes not only in breast but also in other tissues like liver, musculoskeletal or upper airway soft tissues.^[1–3]^ In breast imaging, several studies have indicated that elastography can help evaluate malignant risk.^[4,5]^ In addition, assessment of breast mass by stiffness has significant diagnostic value.^[6]^

Elastography can be classified into 2 systems according to the methods of applying strain: strain elastography and shear wave elastography (SWE).^[7]^ Strain elastography is based on some form of tissue deformation by manual compression of hand pressure and release. Although there is little room for variation in interpreting elastography results, the agreement of measured value in repeated tests can be challenging.^[8]^

On the other hand, SWE uses acoustic impulse to induce slow-moving lateral waves within the tissue. Shear waves travel at different speeds according to the tissue stiffness. On the basis of the measurement of this variable velocity, a “stiffness map” can be created, and the minimum, mean and maximum elasticity values in a region of interest (ROI) can also be identified. In addition, consistent tissue deformation could be induced using a consistent acoustic impulse that is generated electronically; this approach could lead to more reproducible results in the same tissue region.^[9]^

Although SWE is regarded a reliable modality in breast US and provides a quantitative measure and dynamic visual display of tissue stiffness, the issue of inter- and intraobserver variability has not been completely resolved. A phantom study by Mun et al^[10]^ documented reproducibility of SWE in targets of 6 to 11 mm. But this study did not focus on the accuracy and reproducibility by the size of the target. Also, several studies have explored the extrinsic factors influencing US elastography, including background tissue and lesion location^[11,12]^; however, there are scarce resources on intrinsic characteristics that affect SWE reproducibility and accuracy. In this regard, we evaluated the accuracy and reproducibility of the measured elasticity in SWE by using elasticity phantom targets with variable sizes (2.5–18 mm) and stiffness.

## 2. Materials and Methods

Approval from an ethics committee or institutional review board was not necessary for this experimental study because a phantom model was used.

### 2.1. Elasticity phantom model

**Figure 1.**
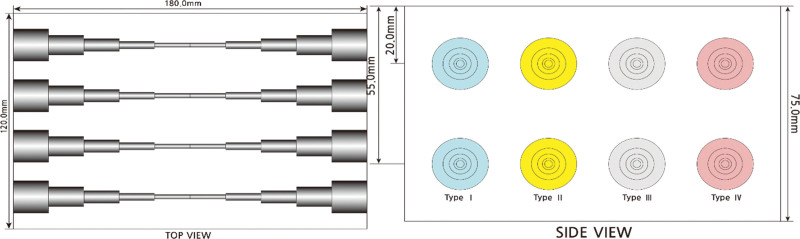
The phantom model (Customized 049A Elasticity QA Phantom, CIRS, Norfolk, VA).

To evaluate the accuracy and reproducibility and to eliminate extrinsic factors such as the thickness of the breast, location of the lesion, and background composition of the breast parenchyma, we used an elasticity phantom model that contained targets with known stiffness and sizes. The phantom we used was a commercially available Elasticity QA phantom model (Customized 049A Elasticity QA Phantom, CIRS, Norfolk, VA) that has 4 types of stepped cylinder-shape lesions with 4 different levels of stiffness (Type1: 11kPa; Type2: 17kPa; Type3: 48kPa; Type4: 80 kPa) and are located at a depth of 2 cm. Each cylinder consists of 5 different parts with different sizes (18, 11, 7, 4, and 2.5 mm); this enables the investigators to evaluate the targets that are located at the same depth and have the same stiffness but with different sizes. Four different types of cylinders with 5 different diameters provided 20 targets. Two pairs of the same lesions were embedded within the phantom, 2 cm from the top and 2 cm from the bottom (Fig. 1).

### 2.2. Data acquisition

Conventional B-mode US, color elastography image, and kPa measurement were obtained for all lesions by using the Aixplorer US system (SuperSonic Imaging, Aix-en-Provence, France) with a 4-15 MHz linear-array transducer. Two radiologists (R1 and R2) examined 20 cross-sectional areas with different diameter in 4 stepped cylinder-shape lesions. This examination yielded a total of 20 round targets with different stiffness levels and sizes.

**Figure 2.**
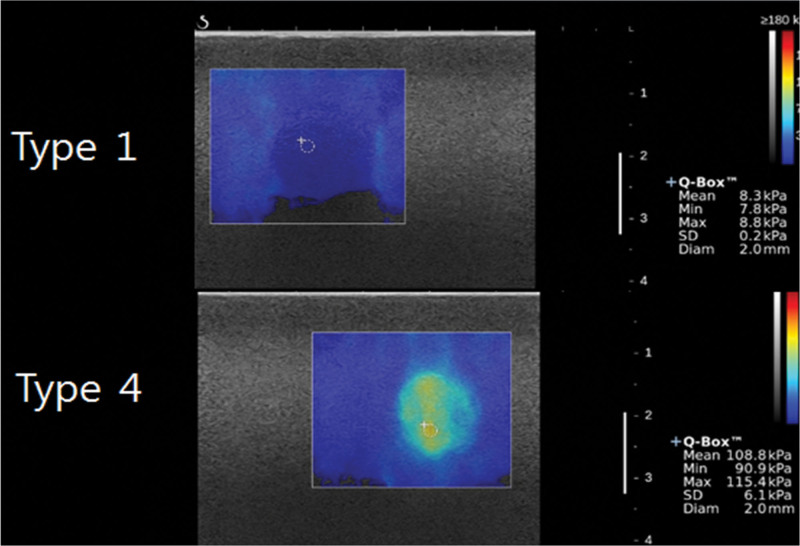
Example of measurements: a 18 mm target.

Elasticity measurement was performed using a 2.0 mm ROI placed at the stiffest area within the targets. The mean, minimum, and maximum elasticity values were measured automatically after placing the ROI. The mean value, which was the most consistent measurement and was not significant affected by subtle location changes in ROI, was used to analyze the accuracy and compare the agreement (Fig. 2).

To evaluate intraobserver reproducibility, the same examinations and measurements were performed twice by each examiner with a 1-week interval. The measurements were independently conducted by the raters.

### 2.3. Data analysis

Inter- and intraobserver reproducibility and the accuracy of measured kPa were evaluated using intraclass correlation coefficient (ICC) analysis. Accuracy was evaluated by comparing the measured elasticity with the known value of the lesion within the phantom. To evaluate any relationship of size and stiffness with the measured kPa, subgroup analysis was performed for inter- and intraobserver reproducibility by size and stiffness. Subgroup analysis could not be conducted on accuracy with different levels of stiffness because comparing the known value and measured kPa did not meet the normality assumption.

Additionally, we performed a Bland-Altman analysis to measure the agreement between raters and evaluate the bias.^[13,14]^ We drew Bland-Altman graphs with the average of 2 measured values on the x-axis and the difference between the 2 measured values on the y-axis. For Bland-Altman graph of reproducibility, the average of 2 measured values was plotted on the x-axis and the difference between the 2 measured values was plotted on the y-axis. For accuracy, a known value was placed on the x-axis to avoid correlation with the y-axis.^[13]^ Fixed bias was represented by a solid line, and expected random error and bias ± 1.96 standard deviation were drawn with dotted lines.

Logistic regression analysis was performed for residual value to check if there was no relationship with target size or stiffness. The residual value was calculated as disagreement between the known value and measured kPa divided by the known value to compensate for the overestimation of the larger numeric gap in high kPa values. In this analysis, stiffness and diameter were set as independent variables and residual values were set as dependent variables. Statistical analysis was performed using SAS version 9.4 (SAS Institute Inc, Cary, NC) and SPSS 24.0. We followed the 2011 Guidelines for Reporting Reliability and Agreement Studies for the analysis and report of reliability and accuracy.^[15]^

## 3. Results

### 3.1. Inter- and intraobserver reproducibility

**Figure 3.**
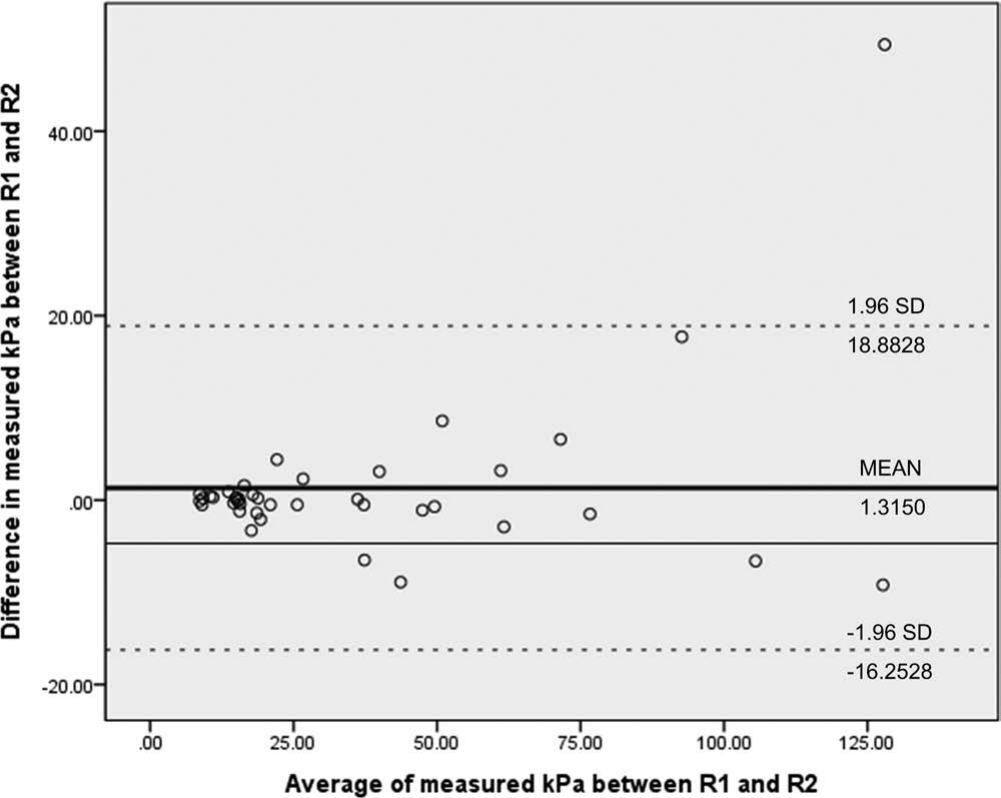
Bland–Altman graph for interobserver reproducibility.

**Figure 4.**
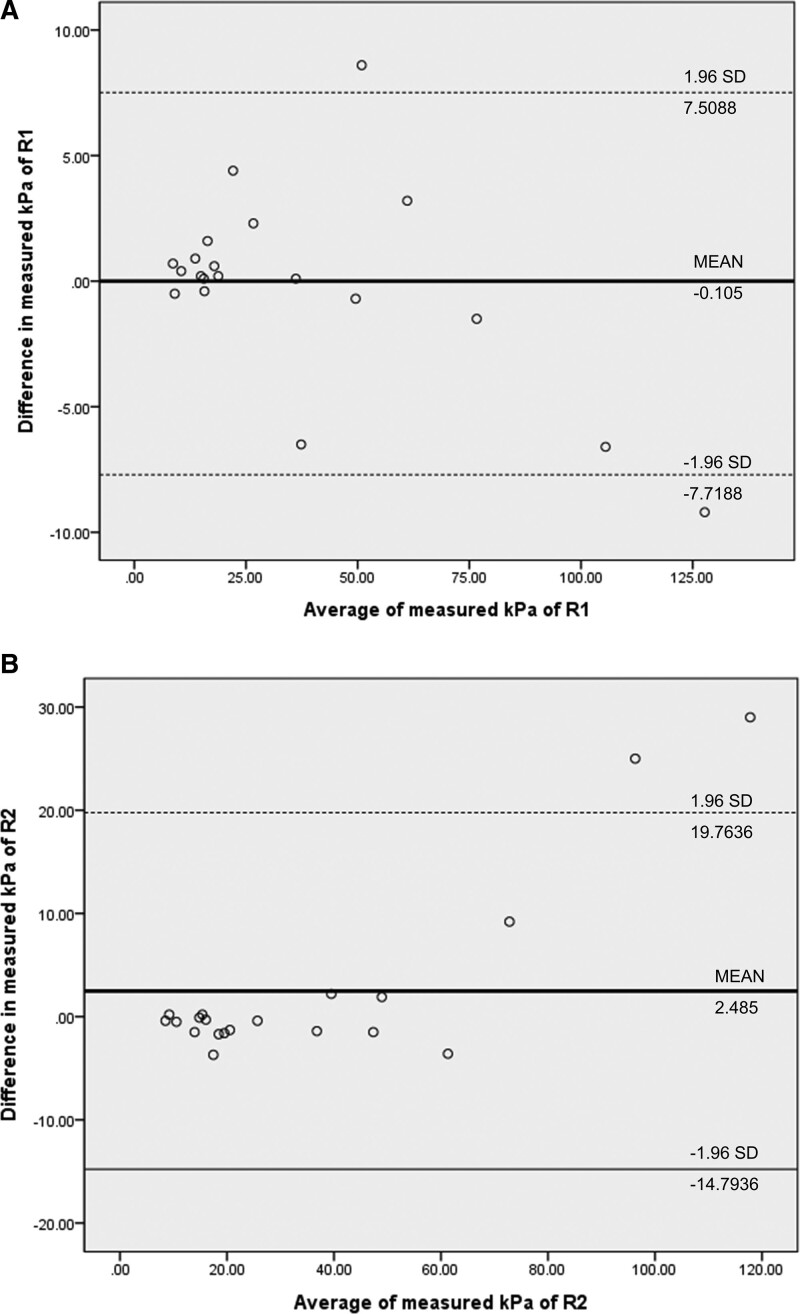
Bland–Altman graph for data measured by R1 (A) and R2 (B).

Interobserver reproducibility for measured kPa value showed excellent agreement in both the initial and repeated examinations (ICC: 0.9742 and 0.9582, respectively) (Table 1). Intraobserver reproducibility showed similar results, with the 2 radiologists obtaining ICC values over 0.9 (Table 1). In subgroup analysis for inter- and intraobserver reproducibility, stiffness and size did not affect SWE reproducibility (Table 2 and 3). The Bland-Altman analysis of the measured kPa between the 2 observers showed a bias of 1.3150 and limits of agreement of 18.8828 to -16.2528; the repeatability coefficient between the 2 raters was 17.5678 (Fig. 3). Data measured by R1 showed a bias of − 0.105 and limits of agreement of 7.5088 to -7.7188; the repeatability coefficient between the 2 raters was 7.6138 (Fig. 4A). Data measured by R2 showed a bias of 2.485 and limits of agreement of 19.7637 to -14.7936; the repeatability coefficient between the 2 raters of 17.2786 (Fig. 4B).

**Table 1:** Interobserver and intraobserver reproducibility of shear wave elastography.

Interobserver reproducibility			
**Wk 1**		**Wk 2**	
ICC	95% CI	ICC	95% CI
0.9742	0.936–0.990	0.9582	0.899–0.983
**Intraobserver reproducibility**			
**Radiologist 1**		**Radiologist 2**	
ICC	95% CI	ICC	95% CI
0.9932	0.984–0.997	0.9294	0.833–0.971

CI = confidence interval, ICC = intraclass correlation coeffient.

**Table 2:** Subgroup analysis of size and stiffness for interobserver reproducibility.

Wk 1				Wk 2		
Diameter (mm)	**ICC**	**95% CI**	***P* value**	**ICC**	**95% CI**	***P* value**
2.5	0.977	0.790–0.998	.002	0.985	0.075–0.999	<.001
4.0	0.865	0.184–0.990	.024	0.990	0.693–0.999	<.001
7.0	0.992	0.919–0.999	.001	0.986	0.865–0.999	.001
11.0	1.000	0.996–1.000	<.001	0.952	0.609–0.997	.006
18.0	0.969	0.723–0.998	.004	0.959	0.635–0.997	.006
Stiffness	**ICC**	**95% CI**	***P* value**	**ICC**	**95% CI**	***P* value**
Type 1	0.995	0.960–0.999	<.001	0.968	0.616–0.997	<.001
Type 2	0.896	0.202–0.989	.003	0.634	-0.143–0.952	.066
Type 3	0.967	0.730—0.996	.001	0.992	0.925–0.999	<.001
Type 4	0.935	0.515–0.993	.004	0.874	0.245–0.986	.006

CI = confidence interval, ICC = intraclass correlation coefficient.

**Table 3:** Subgroup analysis of size and stiffness for intraobserver reproducibility.

R1				R2		
Diameter (mm)	**ICC**	**95% CI**	***P* value**	**ICC**	**95% CI**	***P* value**
2.5	0.966	0.696–0.998	.003	0.989	0.589–0.999	<.001
4.0	0.965	0.556–0.998	.002	0.936	0.435–0.996	.006
7.0	0.994	0.939–1.000	<.001	0.991	0.892–0.999	<.001
11.0	0.997	0.971–1.000	<.001	0.973	0.762–0.998	.003
18.0	0.996	0.953–1.000	<.001	0.903	0.307–0.993	.019
Stiffness	**ICC**	**95% CI**	***P* value**	**ICC**	**95% CI**	***P* value**
Type 1	0. 986	0.869–0.999	<.001	0.814	0.160–0.978	.020
Type 2	0.884	0.376–0.987	.010	0.993	0.938–0.999	<.001
Type 3	0.970	0.752–0.997	.001	0.624	-0.135–0.949	.053
Type 4	0.986	0.888–0.998	<.001	0.987	0.910–0.999	<.001

CI = confidence interval, ICC = intraclass correlation coefficient.

### 3.2. Accuracy

**Figure 5.**
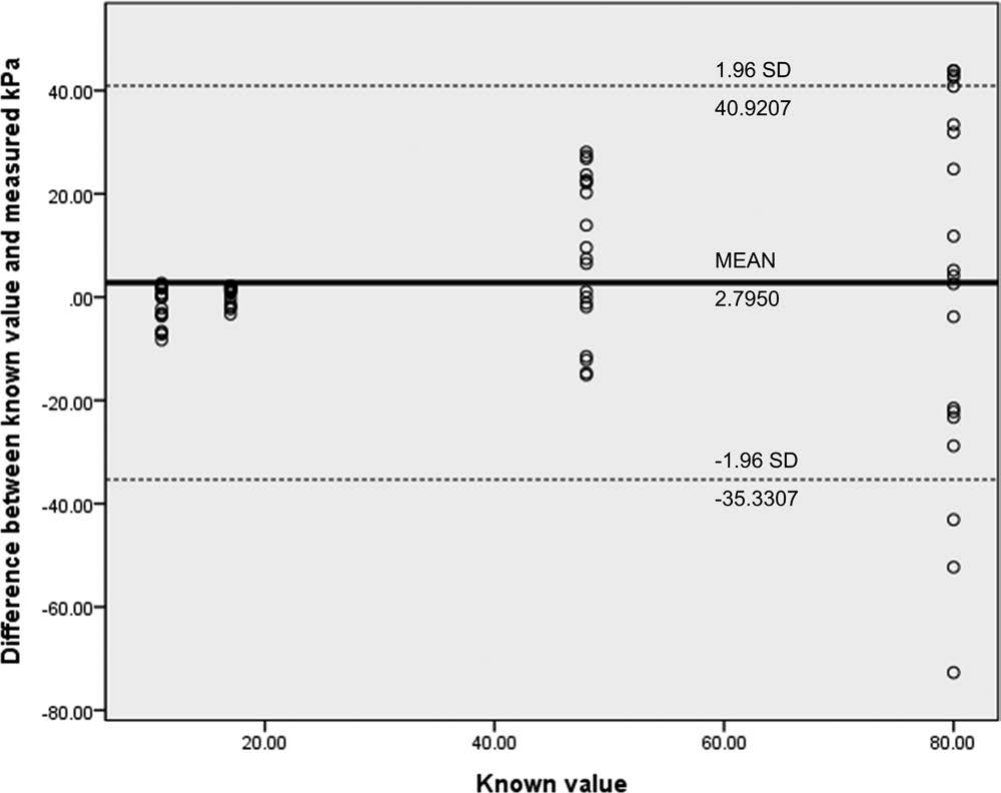
Bland–Altman graph for the agreement of measured values with known value.

The overall accuracy of the measured kPa in SWE was very high (ICC: 0.8049). Targets with a size of 2.5 mm showed low accuracy (ICC: 0.4378, confidence interval (CI): -0.458 to 0.947). Accuracy was higher for targets with a size of 7 and 11 mm (ICC: 0.9832, CI: 0.704–0.999; ICC: 0.9656, CI 0.684–0.998). Targets with a size of 18 mm had lower accuracy than targets with size of 7 or 11 mm (ICC: 0.8487, CI: 0.129–0.989). In the subgroup analysis, targets with a size of 4 mm or less showed significantly lower accuracy (ICC: 0.542, CI: -0.074 to 0.882) than targets of other size (Table 4). The Bland-Altman analysis of the measured kPa and known stiffness value showed a bias of 2.7950 and limits of agreement of 40.9207 to -35.3307; the repeatability coefficient between the 2 raters was 38.1257 (Fig. 5).

**Table 4:** Accuracy of measured kPa of shear wave elastography depending on the target size.

Diameter (mm)	ICC	95% CI	*P* value
Total	0.8049	0.573–0.918	<.001
2.5	0.4378	-0.458–0.947	.211
4.0	0.7062	-0.148–0.977	.069
7.0	0.9832	0.704–0.999	.001
11.0	0.9656	0.684–0.998	.004
18.0	0.8487	0.129–0.989	.030
Subgroups			
2.5–4.0	0.542	-0.074–0.882	.036
7.0–18.0	0.897	0.695–0.969	<.001

CI = confidence interval, ICC = intraclass correlation coefficient.

### 3.3. Residual analysis

**Figure 6.**
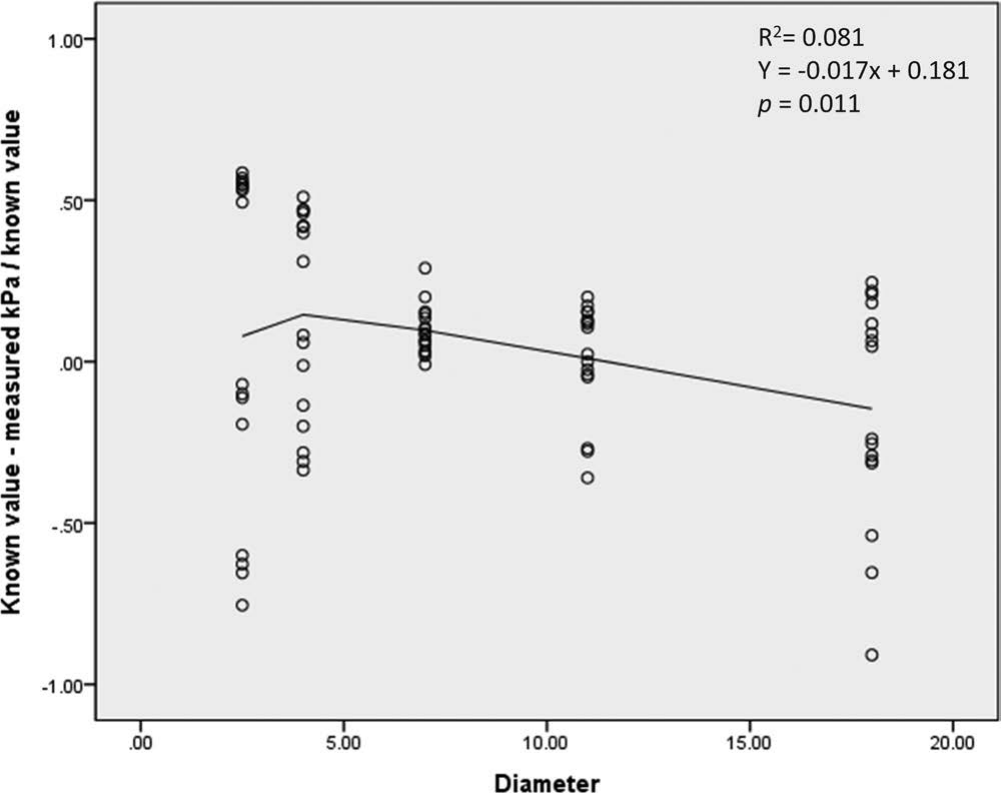
Relationship of residual values and target size.

**Figure 7.**
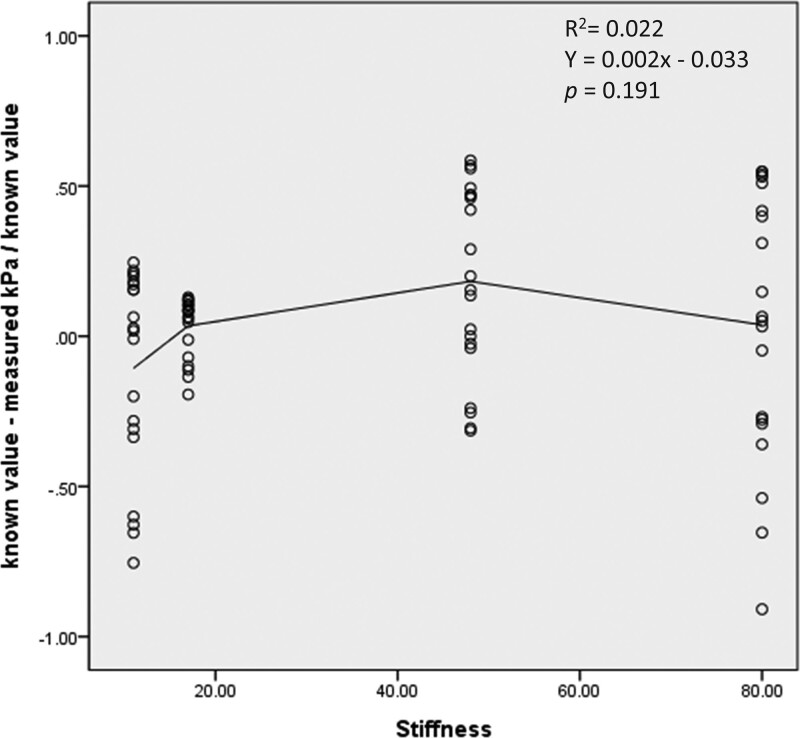
Relationship of residual values and stiffness.

Logistic regression analyses were performed on residual values for both size and stiffness. Both size and stiffness did not show a linear relationship with residual values, and their *R*^2^ were 0.081 and 0.022, respectively (Figs. 6 and 7). In the regression analysis for size and residual values, the coefficient of the regression was -0.017, and the constant of the regression was 0.181 (*P* = .011). In the regression analysis for stiffness and residual values, the coefficient of the regression measured 0.002, and the constant of the regression was -0.033 (*P* = .191).

## 4. Discussion

According to our study results, SWE demonstrated high reproducibility, with both inter- and intraobserver ICC measuring over 0.9 and the statistical significance of p measuring <0.001. Cosgrove et al^[11]^ reported the excellent intraobserver reproducibility of the SWE of palpated breast masses in all diameter, areas, and perimeters and the good agreement of measured kPa (ICC: 0.94, 95% CI: 0.94-0.95; ICC: 0.87, 95% CI: 0.85-0.88). Hong et al^[16]^ reported similar results on the inter- and intraobserver reproducibility of SWE (ICC: 0.879 and 0.803, retrospectively) for the mean value of measured kPa in patients with breast lesions. Park et al^[12]^ reported that the ICC of intraobserver reproducibility was 0.789 with SWE in malignant breast masses. In line with previous studies, the current study supports the excellent reproducibility of SWE in targets with various sizes and elasticity. In this study, a phantom model with a homogenous background was used to avoid confounding effects from various tissue traits in human subjects. The higher reproducibility of our study compared with those of previous studies can be attributed to complete control of extrinsic factors because heterogeneous background breast parenchyma, which is surrounded by mixed fatty and glandular tissue, is reported to affect the reproducibility of elastography.^[12]^

Our accuracy for measuring kPa was high, and the ICC was 0.8049. The stiffness of the targets did not have significant relationship with the accuracy of measured elasticity in the SWE. However, the size showed different results. In small lesions, particularly those measuring 4 mm or less, the agreement between measured kPa and true value was lower than the mean ICC value. The degree of agreement increased in targets between 7 and 11 mm, and slightly decreased in larger target of 18 mm. In the subgroup analysis, the diagnostic accuracy was significantly lower for targets that were 4 mm in size or smaller. There was no linear relationship between the stiffness and accuracy of measured elasticity. Therefore, the careful interpretation of SWE is required, and modalities other than elastography should be considered together for the exact characterization and categorization of the lesions in small indeterminate breast lesions that are 4 mm in size or smaller.

Elastography provides additive diagnostic value not only in differentiating benign or malignant breast lesions but also in assessing the BI-RADS (Breast Imaging Reporting and Data System) category of screening US-detected asymptomatic breast lesions. In combinations with B-mode US, elastography helps downgrade of BI-RADS 4A lesions into BI-RADS 3 or BI-RADS 3 lesions to BI-RADS 2, thus reducing unnecessary breast biopsy or short-term follow-up.^[17]^ Given the promising results, particularly in indeterminate breast lesions, elastography is in the process of being incorporated into routine clinical practice, particularly in evaluating screening-detected, asymptomatic, small breast lesions.

Unlike strain elastography, which requires operator proficiency for stable and accurate results, SWE provides a quantitative value for the stiffness of breast lesions with less hurdle for reaching proficiency.^[18]^ This suggests that SWE provides less operator-dependent quantitative measurement of stiffness in breast lesions. Considering its clinical usefulness, it is important to note that the size of the lesion may affect accurate evaluation of SWE. Along with previous studies reporting high sensitivity and specificity of diagnosis with the use of SWE, the current study provides evidence for the high accuracy and reproducibility of SWE in lesions with various size and stiffness levels, with extra caution needed for smaller lesions.

This study has several limitations. Given that this study used a phantom that has targets with known elasticity values, radiologists who performed SWE examinations could not be blinded. In addition, high reproducibility and accuracy in the phantom may not be in accordance with human subjects, which have heterogeneous background surrounding the lesions and have lesions at different locations. However, previous studies with human subjects have reported similar results,^[11,12,16]^ thus supporting the validity of our study.

We quantitatively measured diagnostic accuracy comparing with the known value in the phantom. In addition, although there have been many studies on diagnostic sensitivity or reproducibility, studies on SWE accuracy are rare, particularly on the influence of intrinsic factors on accuracy. We believe that the results of the present study on the accuracy and association of intrinsic factors with the accuracy of SWE would help establish solid grounds for the diagnostic use of elastography in breast imaging.

In conclusion, SWE shows excellent reproducibility in phantom targets with different sizes and stiffness. The overall accuracy of SWE is good regardless of stiffness but it is questionable in lesions smaller than 4 mm. Therefore, extra caution should be taken when interpreting test results for such lesions.

## Authors’ contributions

**Conceptualization:** Eun Young Ko.

**Data curation:** Boo-Kyung Han.

**Investigation:** Eun Sook Ko.

**Methodology:** Ji Soo Choi.

**Resources:** Haejung Kim, Eun Young Ko.

**Supervision:** Eun Young Ko.

**Writing – original draft:** Harim Kim.

**Writing – review & editing:** Harim Kim.
